# Supplementary material for: Content-rich biological network constructed by mining PubMed abstracts
Source: BMC Bioinformatics. 2004 Oct 8;5:147. doi: 10.1186/1471-2105-5-147 (PMC528731; doi:10.1186/1471-2105-5-147)
Supplement: Additional File 2 — The original results of the above study (non-essential files are deleted to keep the file size under the limit set by BMC bioinformatics). [file 1471-2105-5-147-S2.bz2 › chilibotAdditionalFile2/dip30/684ID9215632E17999/html/CRP_CYTR.html]

 


 **CRP** and **CYTR** 
  
Found 51 abstracts in PubMed, retrieved 30.  
 

 What does Google say? 
 PDF only 
| .edu only 

---

**Interactive relationship** (e.g. stimulation, inhibition, etc)

**Neutral relationship**- Role of protein protein bridging interactions on cooperative assembly of DNA bound  **CRP**   **CytR**   **CRP**  complex and regulation of the Escherichia coli  **CytR**  regulon.  Ref: 12667072 Biochemistry, 2003
- DNA induced changes were observed also in the region involved in  **CRP**   **CytR**  interaction and in  **CRP**  intersubunit contact regions.  Ref: 10388556 J Mol Biol, 1999
- Binding to these multiple sites yields both cooperative and competitive interactions between  **CytR**  and  **CRP** .  Ref: 8969182 J Biol Chem, 1996
- The latter is stabilized by cooperative interactions between  **CRP**  and  **CytR** .  Ref: 8969182 J Biol Chem, 1996
- Transcription initiation from  **CytR**  regulated promoters requires cAMP  **CRP** .  Ref: 8596434 Mol Microbiol, 1995
- The cAMP receptor protein  [ **CRP** ]   **CRP**  and the LacI related  **CytR**  antiactivator bind cooperatively to adjacent DNA sites at or near promoters, an interaction that involves direct protein contacts.  Ref: 9215632 Cell, 1997
- We suggest that the transition from repression to activation involves a switch in the protein protein interactions made by cAMP  **CRP**  from  **CytR**  to RNAP.  Ref: 9086266 J Mol Biol, 1997
- In the presence of  **CytR**  the two regulators bind cooperatively to cddP forming a nucleoprotein complex in which cAMP  **CRP**  binds to  **CRP**  1 and  **CRP**  3 and  **CytR**  occupies the sequence between these sites.  Ref: 1327747 EMBO J, 1992
- The  **CytR**  repressor and the cAMP receptor protein  [ **CRP** ]   **CRP**  bind cooperatively to several promoters in Escherichia coli to repress transcription initiation.  Ref: 9155036 EMBO J, 1997
- **CytR**  controls the balance between  **CRP**  mediated recruitment and activation of RNA polymerase and transcriptional repression.  Ref: 12667072 Biochemistry, 2003
- In E. coli repression of transcription initiation by the  **CytR**  protein relies on  **CytR**  DNA interactions as well as on interactions between  **CytR**  and the cAMP  **CRP**  activator complex.  Ref: 8451188 Nucleic Acids Res, 1993
- Identification of the subunit of cAMP receptor protein  [ **CRP** ]   **CRP**  that functionally interacts with  **CytR**  in  **CRP**   **CytR**  mediated transcriptional repression.  Ref: 10766824 J Biol Chem, 2000
- When acting from a site close to the DNA target for RNA polymerase,  **CytR**  interacts with  **CRP**  to repress transcription.  Ref: 8816767 Proc Natl Acad Sci U S A, 1996
- Transcription is activated by  **CRP**  and repressed by a multi protein  **CRP** . **CytR** . **CRP**  complex.  Ref: 8969182 J Biol Chem, 1996
- However, cooperativity also means that  **CytR**  can recruit  **CRP**  and, by doing so, can act as a co activator.  Ref: 10347150 J Biol Chem, 1999
- The three most upstream rot promoters are activated by the global gene regulatory cAMP  **CRP**  complex and negatively regulated by the  **CytR**  repressor protein.  Ref: 7715459 Mol Microbiol, 1994
- Analysis of  **CRP**   **CytR**  interactions at the Escherichia coli udp promoter.  Ref: 8626289 J Bacteriol, 1996
- Thus, cAMP  **CRP**  induces conformational changes in  **CytR**  so that the repressor fits the natural targets.  Ref: 9155036 EMBO J, 1997
- The central role of cooperativity in regulation is highlighted by the fact that binding of the inducer, cytidine, to  **CytR**  is coupled to  **CytR**   **CRP**  cooperativity.  Ref: 10347150 J Biol Chem, 1999
- Here too, we find that  **CytR**  both cooperates and competes for  **CRP**  binding.  Ref: 10347150 J Biol Chem, 1999
- Transcription is repressed by a three protein complex cAMP receptor protein  [ **CRP** ]   **CRP**   **CytR**   **CRP**  that is stabilized by cooperative interactions between  **CRP**  and  **CytR** .  Ref: 10347150 J Biol Chem, 1999
- The repression region is chemically complementary to the characterized region on  **CytR**  that is essential for protein protein communication to  **CRP** .  Ref: 10320573 Mol Microbiol, 1999
- At promoters of the Escherichia coli  **CytR**  regulon, the cAMP receptor protein  [ **CRP** ]   **CRP**  interacts with the repressor  **CytR**  to form transcriptionally inactive  **CRP**   **CytR**  promoter or  **CRP**  2  **CytR**  promoter complexes.  Ref: 10766824 J Biol Chem, 2000
- Repression takes place by the formation of a  **CytR**  cAMP  **CRP**  DNA nucleoprotein complex consisting of the two cAMP  **CRP**  molecules and  **CytR**  bound in between.  Ref: 7715459 Mol Microbiol, 1994
- Here, using oriented heterodimer analysis, we show that only one subunit of the  **CRP**  dimer, the subunit proximal to  **CytR** , functionally interacts with  **CytR**  in  **CRP**   **CytR**  promoter and  **CRP**  2  **CytR**  promoter complexes.  Ref: 10766824 J Biol Chem, 2000
- These observations can be reconciled in a competition model for  **CytR**  regulation in which  **CytR**  and RNAP compete for cooperative binding with cAMP  **CRP**  to the promoters in vivo.  Ref: 9086266 J Mol Biol, 1997
- These experiments show that the site of action of  **CytR**  can be specified by protein protein interactions to cAMP  **CRP** .  Ref: 8221894 Cell, 1993
- Transcription from cistrons of the Escherichia coli  **CytR**  regulon is activated by E. coli cAMP receptor protein  [ **CRP** ]   **CRP**  and repressed by a multiprotein complex composed of  **CRP**  and  **CytR** .  Ref: 9202008 J Biol Chem, 1997
- Similar extended arrays, but with different arrangements of overlapping  **CytR**  and  **CRP**  sites, are found at the other  **CytR**  regulated promoters.  Ref: 8969182 J Biol Chem, 1996
- Repression by  **CytR**  depends on the formation of nucleoprotein complexes in which  **CytR**  binds cooperatively to the DNA with one or two cAMP  **CRP**  complexes.  Ref: 9086266 J Mol Biol, 1997
- The  **CytR**  repressor protein relies on protein protein interactions to the cAMP  **CRP**  complex to bind its operators with sufficiently high affinity to repress transcription.  Ref: 8764393 J Mol Biol, 1996
- By combining genetic studies and footprinting analyses, we demonstrate that regulated expression of the  **CytR**  controlled cdd promoter requires three  **CRP**  binding sites a high affinity site  **CRP**  1 and two overlapping low affinity sites  **CRP**  2 and  **CRP**  3 centred at positions 4 1 and 93, respectively.  Ref: 1327747 EMBO J, 1992
- The gene encoding the periplasmic cyclophilin homologue, PPIase A, in Escherichia coli, is expressed from four promoters, three of which are activated by the cAMP  **CRP**  complex and negatively regulated by the  **CytR**  repressor.  Ref: 7715459 Mol Microbiol, 1994
- Repression by  **CytR**  depends on the formation of nucleoprotein complexes in which the  **CytR**  repressor and the cAMP  **CRP**  activator complex bind co operatively to the DNA.  Ref: 8596434 Mol Microbiol, 1995
- Maximal repression by the  **CytR**  protein depends on the formation of nucleoprotein complexes in which  **CytR**  interacts with DNA and with cAMP cAMP receptor protein  [ **CRP** ] .  Ref: 8221894 Cell, 1993
- Here, the quaternary structure of  **CytR**  and the mechanism underlying the cooperative binding of  **CytR**  and cAMP  **CRP**  have been analyzed.  Ref: 8764393 J Mol Biol, 1996
- In addition, the  **CytR**  repressor triggers a DNA repositioning of a cAMP  **CRP**  complex in the 35 region upon binding to its operator.  Ref: 8596434 Mol Microbiol, 1995
- Here, we demonstrate that in order to establish  **CytR**  regulation in a cAMP  **CRP**  dependent class II promoter with a single  **CRP**  site  **CRP**  site centred around position 40.5 in which the  **CytR**  operator is located upstream of the  **CRP**  site, high affinity binding sites for both regulators are required.  Ref: 9086266 J Mol Biol, 1997
- Specifically, in class II promoters with a single  **CRP**  site, the efficiency of  **CytR**  regulation was found to correlate inversely with cAMP  **CRP**  independent promoter activity.  Ref: 9086266 J Mol Biol, 1997
- We have suggested that this variability affects the flexibility of  **CytR**  DNA contacts, thereby affecting the critical protein protein interactions between  **CytR**  and the cAMP receptor protein  [ **CRP** ]   **CRP**  that underlie differential repression and activation of  **CytR**  regulated genes.  Ref: 11866516 J Mol Biol, 2002
- Moreover, mutant cdd promoters in which  **CRP**  2 and  **CRP**  3 have been deleted are partially regulated by  **CytR** , and cAMP  **CRP**  and  **CytR**  still bind cooperatively to these promoters.  Ref: 1327747 EMBO J, 1992
- In the CRP2  **CytR**  complex all protein DNA phosphate contacts at  **CRP**  1 and  **CRP**  2 are retained, and in addition two new minor groove contacts, ascribed to phosphate  **CytR**  interactions, are observed at 60 between the  **CRP**  sites.  Ref: 8394345 J Biol Chem, 1993
- Finally, the results show that  **CytR**  and RNA polymerase are rivals that compete for binding with  **CRP**  at deoP2.  Ref: 8394345 J Biol Chem, 1993
- In the deoP2 promoter of Escherichia coli, a transcription activator, cAMP  **CRP** , binds at two sites, centered at 41.5 and 93.5 from the start site of transcription, while a repressor,  **CytR** , binds to a space between the two cAMP  **CRP**  complexes.  Ref: 11574471 EMBO J, 2001
- **CytR**  cAMP  **CRP**  nucleoprotein formation in E. coli the  **CytR**  repressor binds its operator as a stable dimer in a ternary complex with cAMP  **CRP** .  Ref: 8764393 J Mol Biol, 1996
- The Escherichia coli cAMP receptor protein  [ **CRP** ]   **CRP**  is essential for the activation and repression of transcription initiation at promoters in the  **CytR**  regulon.  Ref: 10320573 Mol Microbiol, 1999
- Rather,  **CytR**  possesses a highly flexible interdomain linker that allows it to form complexes with  **CRP**  at promoters with quite different architecture.  Ref: 9466254 Mol Microbiol, 1998
- Analysis of the nupC promoter region revealed the presence of at least two putative  **CRP**  binding sites, centred at 40bp and 89bp, which probably flank a  **CytR**  binding site.  Ref: 8022285 Mol Microbiol, 1994
- In this system, the cAMP receptor protein  [ **CRP** ]   **CRP**  assists the binding of RNA polymerase as well as a specific negative regulator,  **CytR** .  Ref: 8816767 Proc Natl Acad Sci U S A, 1996
- Our data confirm and extend the view that  **CytR**  is a highly flexible DNA binder that can adapt many different conformations for co operative binding with  **CRP** .  Ref: 9466254 Mol Microbiol, 1998
- In  **CytR**  regulated promoters in Escherichia coli, the cAMP  **CRP**  complex acts as a transcriptional activator as well as a co repressor for the  **CytR**  protein.  Ref: 9086266 J Mol Biol, 1997
- Based on recent results about  **CRP**  mediated regulation of transcription initiation and the finding that  **CRP**  possesses discrete surface exposed patches for protein protein interaction with RNA polymerase and  **CytR** , a molecular model for this dual regulation is discussed.  Ref: 8816767 Proc Natl Acad Sci U S A, 1996
- Most strikingly,  **CytR**  can adopt widely different conformations that are equally favored energetically for complex formation with cAMP  **CRP** .  Ref: 9155036 EMBO J, 1997
- The  **CytR**  anti activator diverges from the other family members by relying on the cooperative DNA binding with the global regulator  **CRP** .  Ref: 10329134 J Mol Biol, 1999
- **CRP**  performs these activities by making direct protein protein interactions to the alpha subunits of RNA polymerase and to the  **CytR**  regulator.  Ref: 10320573 Mol Microbiol, 1999
- Cooperative interactions between  **CytR** , when bound to an operator CytO located upstream of a  **CytR**  regulated promoter, and  **CRP** , when bound to flanking tandem promoters, are critical to the regulatory role of  **CytR** .  Ref: 12667072 Biochemistry, 2003
- Transcription initiation at  **CytR**  regulated promoters in Escherichia coli is controlled by a combinatorial regulatory system in which the cAMP receptor protein  [ **CRP** ]   **CRP**  functions as both an activator and a co repressor.  Ref: 1327747 EMBO J, 1992
- Recently, we showed that  **CytR**  binds specifically to multiple sites in the E. coli deoP promoter, thereby providing competition for  **CRP**  binding to  **CRP**  operator site 1 CRP1 and CRP2 as well as cooperativity.  Ref: 10347150 J Biol Chem, 1999
- Using a modified Ferguson analysis in which protein DNA complexes are separated in a non denaturing gel system, we show that  **CytR**  binds its operators as a dimer alone as well as in a ternary complex with cAMP  **CRP** .  Ref: 8764393 J Mol Biol, 1996
- The unlinked operons that comprise the Escherichia coli  **CytR**  regulon are controlled coordinately through interactions between two gene regulatory proteins, the cAMP receptor protein  [ **CRP** ]   **CRP**  and the cytidine repressor  **CytR** .  Ref: 12667072 Biochemistry, 2003
- Furthermore,  **CytR**  proteins deleted for the DNA binding domain repress deoP2 in vivo and interact with deoP2 in vitro in a strictly cAMP  **CRP**  dependent fashion.  Ref: 8221894 Cell, 1993
- The cAMP  **CRP**  complex and the  **CytR**  anti activator bind co operatively to the promoter region forming a repression complex that overlaps the sigmaE dependent P3 promoter and the sigma70 dependent P4 and P5 promoters.  Ref: 9767576 Mol Microbiol, 1998
- Unlike the other members of this protein family,  **CytR**  binds with only modest affinity to its operators and transcription repression thus relies on the formation of nucleoprotein complexes with the cAMP  **CRP**  complex.  Ref: 9466254 Mol Microbiol, 1998
- The  **CytR**  protein was shown to act as a transcription repressor or activator, depending on the configuration of the promoter and on the relative location and quantity of binding sites for  **CytR**  and  **CRP**  proteins.  Ref: 9777353 Genetika, 1998
- Moreover, the results provide insight into the mechanism by which  **CytR**  might prevent  **CRP**  mediated transcription.  Ref: 10320573 Mol Microbiol, 1999
- The Escherichia coli  **CytR**  regulator belongs to the LacI family of sequence specific DNA binding proteins and prevents  **CRP**  mediated transcription in the  **CytR**  regulon.  Ref: 9466254 Mol Microbiol, 1998
- The stimulation of udp expression by  **CytR**  in certain mutants may reflect its stabilization of cAMP  **CRP**  binding to target DNA and illustrates that only modest evolutionary changes could allow particular multiprotein complexes to serve as either repressors or transcriptional activators.  Ref: 8626289 J Bacteriol, 1996

**Non-interactive relationship** (e.g. studied together, co-existance, homology, etc.)

- In the absence of  **CytR** , cAMP  **CRP**  interacts at one set of sites  **CRP**  1 and  **CRP**  2 and both of these binding sites are required for full promoter activation.  Ref: 1327747 EMBO J, 1992
- To understand the combinatorial control mechanism at deoP2, we have used quantitative footprint and gel shift analysis of  **CRP**  and  **CytR**  binding to evaluate the distribution of ligation states.  Ref: 8969182 J Biol Chem, 1996
- Despite only weak association between  **CytR**  and  **CRP**  in solution, our results demonstrate the formation of a relatively stable complex in which the Core domain forms a protein bridge between tandem  **CRP**  dimers when bound to either udpP or deoP2.  Ref: 12667072 Biochemistry, 2003
- Here, we identify a collection of amino acid substitutions in  **CytR**  that reestablish protein protein communication to mutant  **CRP**  proteins specifically defective in cooperative binding with wild type  **CytR** .  Ref: 9215632 Cell, 1997
- However, this cytidine mediated effect varies among promoters, suggesting that coupling between cytidine binding to  **CytR**  and  **CytR**   **CRP**  association is sensitive to promoter structure.  Ref: 12667072 Biochemistry, 2003
- Our results established that residues 1 0 08 and 110 are essential for the interaction with  **CytR**  and confirmed that activating region 2 of  **CRP**  is made up of residues 1 1 and 101.  Ref: 10320573 Mol Microbiol, 1999
- We discuss these novel concepts in the context of the  **CRP**   **CytR**  regulatory system, as well as the structural and functional implications for multiprotein DNA complex formation in general.  Ref: 9155036 EMBO J, 1997
- Here, we have used quantitative footprint and mobility shift analysis to investigate  **CRP**  and  **CytR**  binding to the E. coli udp promoter.  Ref: 10347150 J Biol Chem, 1999
- Furthermore, our results permit the construction of a three dimensional structure of the higher order nucleoprotein complex formed by  **CytR**  and  **CRP** .  Ref: 9215632 Cell, 1997
- Protein induced fit the  **CRP**  activator protein changes sequence specific DNA recognition by the  **CytR**  repressor, a highly flexible LacI member.  Ref: 9155036 EMBO J, 1997
- These findings provide clues to an understanding of how cAMP  **CRP**  and  **CytR**  interact at a structurally diverse set of promoters.  Ref: 1327747 EMBO J, 1992
- Identification of the nucleotide sequence recognized by the cAMP  **CRP**  dependent  **CytR**  repressor protein in the deoP2 promoter in E. coli.  Ref: 8451188 Nucleic Acids Res, 1993
- Here, in vitro selection experiments have been used to examine the DNA binding characteristics of  **CytR** , by itself and when co binding with cAMP  **CRP** .  Ref: 9155036 EMBO J, 1997
- The cAMP  **CRP**   **CytR**  nucleoprotein complex in Escherichia coli two pairs of closely linked binding sites for the cAMP  **CRP**  activator complex are involved in combinatorial regulation of the cdd promoter.  Ref: 1327747 EMBO J, 1992
- The mechanisms for the cAMP  **CRP**  mediated transcription activation and  **CytR**  mediated transcription repression were investigated in vitro using purified components.  Ref: 11574471 EMBO J, 2001
- The effect of the competition at this promoter is to negate the role of  **CytR**  in recruiting  **CRP** .  Ref: 10347150 J Biol Chem, 1999
- The structure of the cAMP  **CRP**   **CytR**  repression complex and the cAMP  **CRP**  RNA polymerase initiation complex at the deoP2 promoter of E. coli have been probed by DNase I and uranyl footprinting.  Ref: 8394345 J Biol Chem, 1993
- These findings show that the bacterium uses positive and negative regulatory modules to differentially control the expression of  **CytR**  and cAMP  **CRP**  regulated genes.  Ref: 8596434 Mol Microbiol, 1995
- We propose that competition and cooperativity in  **CytR**  and  **CRP**  binding are important to differential regulation of these promoters.  Ref: 8969182 J Biol Chem, 1996
- Dual function regulators the cAMP receptor protein  [ **CRP** ]  and the  **CytR**  regulator can act either to repress or to activate transcription depending on the context.  Ref: 8816767 Proc Natl Acad Sci U S A, 1996
- In mutants with an improved 10 region, a partial relief from the control of the cAMP  **CRP**  transcription activation complex occurred, and the negative  **CytR**  repressor regulation was reduced.  Ref: 12391883 Genetika, 2002
- Our results support the model in which repression normally involves the binding of  **CytR**  to cAMP  **CRP**  to form a complex which binds to specific sites in the udp promoter, without direct interaction between  **CytR**  protein and a specific operator DNA sequence, and in which induction by specific inducer cytidine involves dissociation of  **CytR**  from cAMP  **CRP**  and the RNA polymerase interaction with cAMP  **CRP**  bound to a site upstream of then transcription start point.  Ref: 8626289 J Bacteriol, 1996
- Binding of cAMP receptor protein  [ **CRP** ]   **CRP**  and  **CytR**  mediates both positive and negative control of transcription from Escherichia coli deoP2.  Ref: 8969182 J Biol Chem, 1996
- On the basis of the regulatory features of the promoters analysed here, we speculate about the advantages offered by the structural complexity of natural  **CytR**  cAMP  **CRP**  regulated promoters.  Ref: 9086266 J Mol Biol, 1997
- Here we show that the Escherichia coli nupG gene contains one  **CytR**  and four  **CRP**  binding sites in the control region.  Ref: 8596434 Mol Microbiol, 1995
- Gene regulatory modules in Escherichia coli nucleoprotein complexes formed by cAMP  **CRP**  and  **CytR**  at the nupG promoter.  Ref: 8596434 Mol Microbiol, 1995
- In particular, the transcription of genes needed for utilization of nucleosides in Escherichia coli is regulated by a repressor protein,  **CytR** , in concert with the cyclic AMP cAMP activated form of cAMP receptor protein  [ **CRP** ]   **CRP** .  Ref: 8626289 J Bacteriol, 1996
- Here we show that, depending on promoter architecture, the  **CRP**   **CytR**  nucleoprotein complex has opposite effects on transcription.  Ref: 8816767 Proc Natl Acad Sci U S A, 1996
- Characterization of promoter recognition complexes formed by  **CRP**  and  **CytR**  for repression and by  **CRP**  and RNA polymerase for activation of transcription on the Escherichia coli deoP2 promoter.  Ref: 8394345 J Biol Chem, 1993
- To investigate the chemical and structural basis for these effects, we investigated how cytidine binding affects association between  **CytR**  and  **CRP**  in solution and how it affects the binding of  **CytR**  deletion mutants lacking the DNA binding HTH domain, with tandem  **CRP**  dimers bound to either udpP or deoP2.  Ref: 12667072 Biochemistry, 2003
- This approach enables us to specify the patch on  **CytR**  s surface that contacts  **CRP** .  Ref: 9215632 Cell, 1997
- Transcription of rpoH, encoding the Escherichia coli heat shock regulator sigma32, is negatively controlled by the cAMP  **CRP**   **CytR**  nucleoprotein complex.  Ref: 9767576 Mol Microbiol, 1998
- A flexible partnership the  **CytR**  anti activator and the cAMP  **CRP**  activator protein, comrades in transcription control.  Ref: 8736525 Mol Microbiol, 1996
- In Process Citation Site directed mutagenesis was conducted in the regulatory region of the Escherichia coli udp gene at promoter sites responsible for binding regulatory proteins  **CRP**  and  **CytR**  as well as RNA polymerase.  Ref: 12391883 Genetika, 2002
- These findings demonstrate that direct coupling between cytidine binding to  **CytR**  and  **CytR**   **CRP**  association does not account for promoter specific effects on cooperativity.  Ref: 12667072 Biochemistry, 2003
- Sequences similar to the sigma 70 promoters P1, P4 and P5, to the sigma E promoter P3 and to boxes DnaA1, DnaA2, cAMP receptor protein  [ **CRP** ]   **CRP**  boxes CRP1, CRP2 and box  **CytR**  present in Escherichia coli K12, were identified in sequences of closely related bacteria such as E.coli, Shigella flexneri, Salmonella enterica serovar Typhimurium, Citrobacter freundii, Enterobacter cloacae and Klebsiella pneumoniae.  Ref: 11139607 Nucleic Acids Res, 2001
- The data presented suggest that the expression of the P2 promoter, unlike that of P1, is not subjected to regulation with participation of the  **CytR**  protein and the cAMP  **CRP**  complex.  Ref: 12068544 Genetika, 2002
- Design of  **CytR**  regulated, cAMP  **CRP**  dependent class II promoters in Escherichia coli RNA polymerase promoter interactions modulate the efficiency of  **CytR**  repression.  Ref: 9086266 J Mol Biol, 1997
- Cooperativity with  **CRP**  at CRP1 is nearly eliminated, but the effect on  **CytR**  CRP2 cooperativity is negligible.  Ref: 10347150 J Biol Chem, 1999
- Our results provide information about the architecture of  **CRP**   **CytR**  promoter and  **CRP**  2  **CytR**  promoter complexes and rule out the proposal that masking of activating region 2 of  **CRP**  is responsible for the transcriptional inactivity of the complexes.  Ref: 10766824 J Biol Chem, 2000
- Thus, these data rule out the possibility that the cooperative DNA binding of  **CytR**  and cAMP  **CRP**  involves induced dimerization of  **CytR** , and they suggest that cytidine interrupts the cooperative binding of  **CytR**  and cAMP  **CRP**  solely by perturbing the protein protein interactions between the two proteins.  Ref: 8764393 J Mol Biol, 1996
- After introduction of mutations affecting binding sites for  **CRP** , the negative effect of the  **CytR**  protein on promoter transcription was fully abolished.  Ref: 12391883 Genetika, 2002
- The mechanism of repression by  **CytR**  proposed in this study is that the cAMP  **CRP**  bound at 41.5 undergoes an allosteric change upon direct interaction with  **CytR**  such that it no longer maintains a productive interaction with the N terminal domain of alpha, but instead acts as a repressor to interfere with RNA polymerase acting on deoP2.  Ref: 11574471 EMBO J, 2001
- Protein protein communication structural model of the repression complex formed by  **CytR**  and the global regulator  **CRP** .  Ref: 9215632 Cell, 1997
- Multiple specific  **CytR**  binding sites at the Escherichia coli deoP2 promoter mediate both cooperative and competitive interactions between  **CytR**  and cAMP receptor protein  [ **CRP** ] .  Ref: 8969182 J Biol Chem, 1996
- However, when co binding with cAMP  **CRP** ,  **CytR**  instead recognizes inverted repeats separated by 10 13 bp, or direct repeats separated by 1 bp.  Ref: 9155036 EMBO J, 1997
- Multiple binding sites for the  **CytR**  and  **CRP**  proteins were identified outside the canonical udp gene promoter.  Ref: 9777353 Genetika, 1998
- In this model, two mutually exclusive ternary complexes can be formed a  **CytR**  cAMP  **CRP**  promoter repression complex and an RNAP cAMP  **CRP**  promoter activation complex.  Ref: 9086266 J Mol Biol, 1997
